# Barriers and drivers to childhood vaccinations in Forcibly Displaced Myanmar Nationals (FDMN)/Rohingya refugees in Cox’s Bazar, Bangladesh: a scoping review

**DOI:** 10.3389/fpubh.2025.1592452

**Published:** 2025-07-18

**Authors:** Zarah Yusuf, Sarah Reda, Johanna Hanefeld, Cath Jackson, Balwinder Singh Chawla, Andreas Jansen, Saskia Lange, Jorge Martinez, Emily Dorothee Meyer, Julia Neufeind, Aarti Shrikrishana Singh, Elisa Wulkotte, Md. Shamsuz Zaman, Basel Karo

**Affiliations:** ^1^London School of Hygiene and Tropical Medicine, London, United Kingdom; ^2^Robert Koch Institute, Centre for International Health Protection, Information Centre for International Health Protection (INIG), Berlin, Germany; ^3^Valid Research Ltd, East Sussex, United Kingdom; ^4^World Health Organization Country Office, Dhaka, Bangladesh; ^5^World Health Organization Emergency Sub-Office, Cox’s Bazar, Bangladesh; ^6^Department of Infectious Disease Epidemiology, Postgraduate Training for Applied Epidemiology (PAE), Robert Koch Institute, Berlin, Germany; ^7^ECDC Fellowship Programme, Field Epidemiology Path (EPIET), European Centre for Disease Prevention and Control (ECDC), Solna, Sweden; ^8^Department of Infectious Disease Epidemiology, Immunization Unit, Robert Koch Institute, Berlin, Germany; ^9^Cox's Bazar Medical College, Cox's Bazar, Bangladesh

**Keywords:** Rohingya, Forcibly Displaced Myanmar Nationals/Rohingya refugees, childhood, vaccination, Cox’s Bazar, review

## Abstract

About one million Forcibly Displaced Myanmar Nationals (FDMN)/Rohingya refugees live in the refugee camps of Cox’s Bazar, experiencing recurring vaccine-preventable disease outbreaks despite established vaccination programs. This scoping review focused on the evidence for individual and context barriers, drivers, and interventions for childhood vaccination uptake of FDMN/Rohingya refugees in Cox’s Bazar. Four databases and grey literature were systematically searched. Theoretical frameworks were used to organize findings. 4,014 records were screened, and 21 articles included. The literature was heterogenous. Barriers and drivers for FDMN/Rohingya refugees receiving vaccination focused on motivation relating to trust, beliefs and fears (19 barriers and drivers in 11 articles), accessibility and information availability (19 barriers and drivers in 11 articles), as well as knowledge and ability (eight barriers and drivers in nine articles), and socio-cultural and gender-related norms and social support (seven barriers and drivers in eight articles). For health service providers facilitating vaccinations, context factors, such as the availability of vaccines and staff, were most frequently identified (13 barriers and drivers in 12 articles). Interventions mostly related to vaccination campaigns and information/education. They often lacked detail and formal evaluations. Future research and interventions on childhood vaccination should consider barriers and drivers for health service providers, the diversity of the camp population, and explore the role of community/religious leaders and gender-related social norms. Additionally, the reporting and evaluation of interventions should be strengthened.

**Systematic review registration:**

https://doi.org/10.17605/OSF.IO/N6D3URL; https://osf.io/n6d3z.

## 1 Introduction

### 1.1 Background

Vaccines are one of the most cost-effective and lifesaving public health measures to date, importantly reducing the burden of vaccine-preventable diseases (VPD) such as poliomyelitis, measles and rubella globally (1). However, they have not been able to reach their full potential in vulnerable groups like refugee populations. The global literature reports that the latter experience higher VPD burden and lower immunisation rates than other populations due to several reasons (2, 3): These include overcrowding in refugee camps and poor water, sanitation, and hygiene (WASH) conditions that facilitate the rapid spread of VPD (3, 4). Multiple challenges exist in accessing and delivering health services (including vaccines) in refugee camps on the context level (5). Vaccine hesitancy and lack of vaccine confidence can cause delay or refusal of vaccinations despite their availability on an individual level (6). This is a common phenomenon globally with reasons including vaccine misinformation, unfamiliarity with or mistrust of health systems, language barriers, and sociocultural differences (7).

One group especially at risk are Forcibly Displaced Myanmar Nationals (FDMN)/Rohingya refugees. In 2017, 700,000 FDMN/Rohingya fled to Cox’s Bazar, Bangladesh, following an increase in longstanding ethnic and religious persecution of Rohingya people in Myanmar (8). They joined approximately 300,000 previously settled Rohingya refugees. Access to health services and vaccinations in Myanmar was found to be highly inequitable for Rohingya with a study showing 60% of Rohingya children arriving in Bangladesh had never previously been vaccinated (9). Densely populated makeshift settlements, coupled with a poor health status and low immunisation coverage led to several VPD outbreaks (4), including the largest reported diphtheria outbreak in refugee settings so far (10).

Denied of citizenship in Myanmar, FDMN/Rohingya refugees are the largest stateless population in the world, currently estimated at just over 1 million people (11, 12). They live, spread over 35 camps, in two subdistricts of Cox’s Bazar, Uhyia and Teknaf (11, 12). The camps vary in size and accessibility, and function as self-organised, independent districts, each divided into blocks and subblocks. The health response including the vaccination program is led by the Health Sector Strategic Advisory Group with representatives from the Government of Bangladesh, United Nations bodies, national and international non-governmental organisations (NGOs) (8). To control and prevent further outbreaks, mass vaccination campaigns were initiated in September 2017, and routine immunisation services established in February 2018 (13). Essential health services including vaccinations are delivered by 93 health posts, 46 primary health care centres and five field hospitals provide essential health services including vaccinations (14). Vaccinations are provided via fixed vaccination sites and community outreach, and follow the Expanded Program on Immunisation for Bangladesh (15). Multiple professional groups are involved in facilitating vaccinations. Community health workers (CHWs) from the refugee and local host population counsel community members and support vaccination provision. Vaccinators are mostly from the local host community and receive special training. The health service provision of each facility is overseen by a health facility manager. Additionally, the community-based Camp-in-Charge (CiC) group in each camp and around 15 Immunisation partners (including BRAC, UNHCR, UNICEF and IOM) support the vaccination program (13). Official numbers for vaccination coverage of Rohingya children in Cox’s Bazar are lacking, and estimates range from 23–78% (16). Modelling studies have suggested that VPD outbreaks are still likely and a threat to FDMN/Rohingya refugees as well as host communities (10, 17).

To increase vaccination coverage, it is essential to understand the complex and multifaceted nature of vaccination behaviours and processes (18). This includes examining both demand and supply-side factors, including assessment of the barriers and drivers for patients or caregivers in receiving vaccinations, as well as the barriers and drivers for health service providers (HSP) in facilitating them (18). A global evidence review found that for HSP working in migrant or refugee settings, numerous barriers to delivering vaccinations exist (19). These may include resource and capacity constraints, cold chain limitations, inaccessibility, insufficient safety for professionals, or lacking knowledge of migrant health care needs and culturally competent care (19).

To date, the available evidence on barriers, drivers and interventions for childhood vaccination for FDMN/Rohingya refugees and HSP in Cox’s Bazar has not been reviewed.

### 1.2 Aim and objectives

The aim of this scoping review was to gain understanding and review the research landscape of childhood vaccinations in FDMN/Rohingya refugees in Cox’s Bazar to inform future research and strategies to increase vaccination uptake.

The objectives were to identify individual and context barriers and drivers to *receiving* childhood vaccination amongst FDMN/Rohingya refugee caregivers and *facilitating* childhood vaccinations by HSP in Cox’s Bazar. Exploring these different perspectives ensured that both the demand and supply side of vaccination coverage are considered (20). A further objective was to identify vaccination interventions that have been recommended or implemented (with or without evaluation), in this population.

## 2 Methods

### 2.1 Scoping review approach

This review was conducted as part of a broader scoping review focusing on childhood vaccinations and COVID-19 protective behaviours (including vaccinations) of FDMN/Rohingya refugees in Cox’s Bazar. This paper presents the childhood vaccination part only. This scoping review followed the methodology of the Joanna Briggs Institute (JBI) (21) based on the Arksey and O’Malley framework for scoping reviews (22). A protocol was uploaded onto the Open Science Framework (OSF) website prior to accessing the data.^1^ The Preferred Reporting Items for Systematic reviews and Meta-Analysis extension for Scoping Reviews (PRISMAScR) guided the reporting (see Supplementary material 1 for PRISMAScR Checklist) (23).

### 2.2 Theoretical background: modified COM-B framework and behaviour change wheel

**Figure 1:**
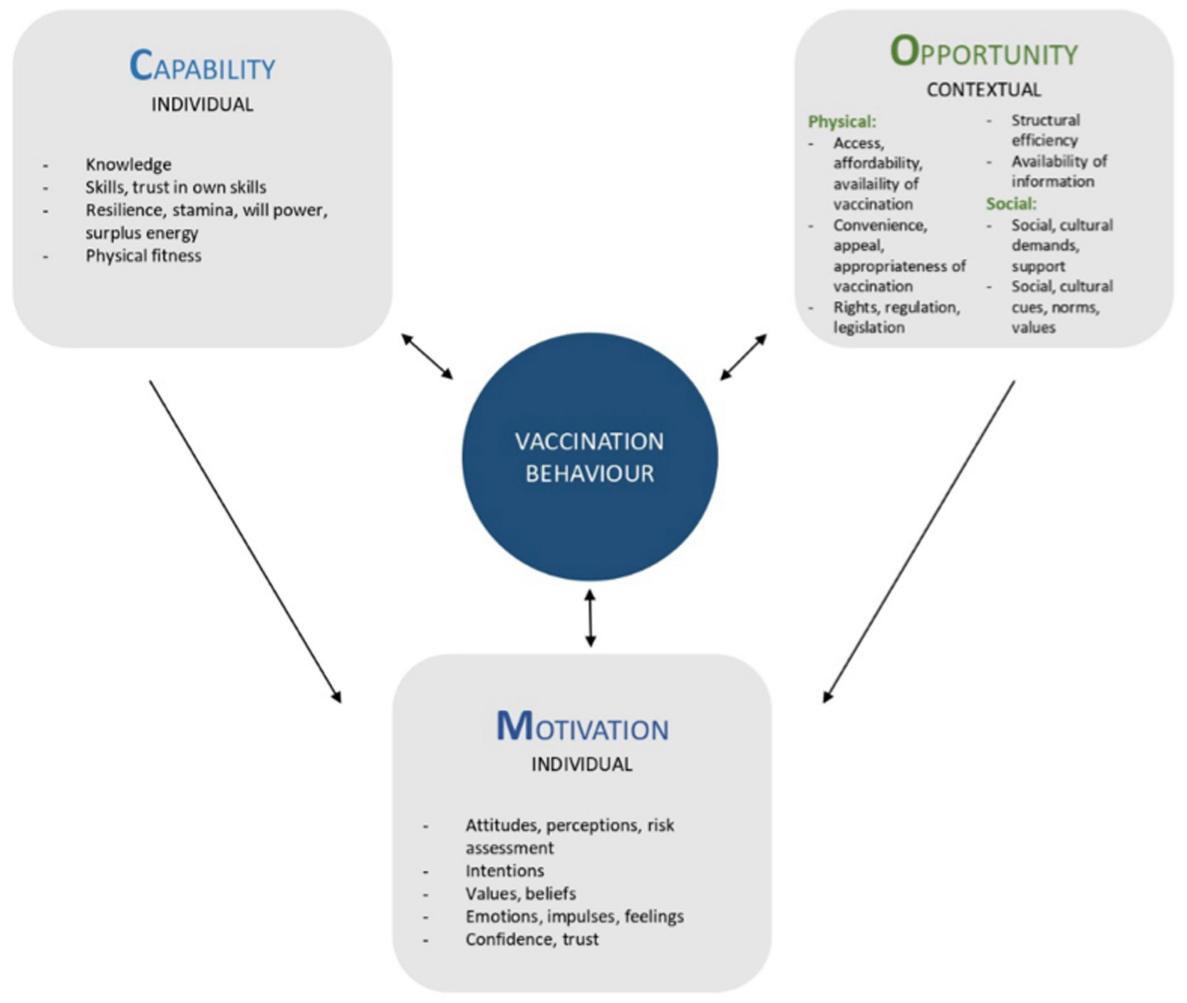
Modified COM-B framework for vaccination (18).

This review was underpinned by the Capability-Opportunity-Motivation-Behaviour (COM-B) framework and Behaviour Change Wheel (BCW) (24), modified for vaccination behaviours (see Figure 1 and Table 1).

**Table 1:** Linking intervention types to COM-factors (20, 24).

Intervention type	Intervention description	COM-factor addressed by the intervention type			
		Capability	Physical opportunity	Social opportunity	Motivation
Information/education	Increasing knowledge or understanding	X			X
Persuasion	Using communication to induce positive/negative feelings or stimulate action				X
Incentivisation	Creating an expectation of a reward				X
Coercion	Creating an expectation of punishment or cost				X
Training	Imparting skills	X	X		X
Restriction	Using rules to reduce the opportunity to engage in the target behaviour or in competing behaviours		X	X	
Environmental restructuring	Changing the physical or social context.		X	X	X
Modelling	Providing an example of people to aspire to or imitate.			X	X

The modified COM-B framework suggests that four inter-linked factors influence vaccination behaviour: Capability (e.g., knowledge, skills), physical opportunity (information, access, health systems), social opportunity (support, norms) and motivation (attitudes, confidence, trust). All factors affect an individual’s motivation for positive vaccination behaviours, with caregivers bringing their children to receive vaccinations and HSP facilitating the provision of vaccinations. The framework’s comprehensive approach, incorporating both individual and context influences (20) on the demand and supply side, was deemed particularly appropriate to categorize the barriers and drivers.

The modified BCW links the four COM factors with eight types of interventions: Information/education, persuasion, incentivisation, coercion, training, restriction, environmental restructuring, and modelling. These intervention types have proved effective at addressing specific COM-factors, and hence ensure that the appropriate interventions are employed (24). Table 1 describes the intervention types and shows which COM-factors they effectively address. The four COM-factors and eight intervention types of the BCW were used throughout this review to organise the barriers, drivers, and intervention data, respectively.

### 2.3 Search strategy and information sources

The electronic databases Ovid MEDLINE, Excerpta Medica database (Embase), Global Health and Web of Science were searched using Boolean operators, keywords, and subject headings. Initial database searches and screening were conducted simultaneously for both topics up to October 2021. In June 2024, an update search was conducted. Details on search strings are provided in Supplementary material 3. Grey literature was searched systematically through the websites Google, *Reliefweb.int* and *Humanitarianresponse.info*. In addition, individual websites from development partners of the Ministry of Health, United Nations (UN) and non-governmental organizations (NGO) in Cox’s Bazar were searched (see Supplementary material 4). A snowball search of reference lists was conducted.

### 2.4 Eligibility criteria

The protocol used the Population, Concept, Context (PCC) framework for inclusion and exclusion criteria (21) as recommended for scoping reviews as per the JBI methodology (see Supplementary material 2).

Childhood vaccinations in this review are defined as vaccinations given to a person aged 0–18 years of age as part of routine or supplemental immunisation programs (such as mass vaccination campaigns).

Regarding the concept of vaccination, two distinct target behaviours were defined. *Receiving vaccinations* refers to FDMN/Rohingya refugee caregivers accepting and accessing vaccination services and bringing their child(ren) for vaccination. *Facilitating vaccinations* involves HSPs educating, enabling, and administering childhood vaccinations.

### 2.5 Study selection

Articles were screened at title/abstract [using Rayyan (25)] and full-text level by two independent investigators (ZY, SR). A third researcher (CJ) was involved when there were disagreements on eligibility.

A data extraction matrix based on the PCC framework was developed in Microsoft Excel, and data on barriers, drivers and interventions were extracted from full texts by one researcher (ZY). This followed a pilot data extraction of three studies in which results were compared with two other researchers for consistency (SR, CJ). Queries were discussed among the researchers and agreed by consensus. Records and data were managed with Endnote and Excel programs.

### 2.6 Data extraction and synthesis

Data synthesis was guided by the principles of textual narrative synthesis (26), for which a table of COM-factors and target behaviours (receiving, facilitating) was developed to organise barriers and drivers. It was also noted whether this was the perspective of FDMN/Rohingya refugees, HSP, or both. Where data permitted, similarities and differences across camps and population groups (e.g., age, gender) were identified. Similarly, a table was developed to organise intervention data into recommended, implemented or evaluated interventions and by intervention types of the BCW (24).

### 2.7 Critical appraisal of evidence sources

Though critical appraisal is optional for scoping reviews (22), it was deemed useful in the context of mapping out the evidence base and assessing the quality of the literature landscape. Three tools were used to critically appraise different article types: The Mixed Method Assessment Tool (MMAT) for research studies (27), the Authority, Accuracy, Coverage, Objectivity, Date, Significance Checklist (AACODS) for grey literature (28), and the JBI Checklist for text and opinion pieces (29). This was done by two researchers independently (ZY, SL). Scores were compared and agreed by consensus (see Supplementary material 6).

## 3 Results

### 3.1 Overview of results

**Figure 2:**
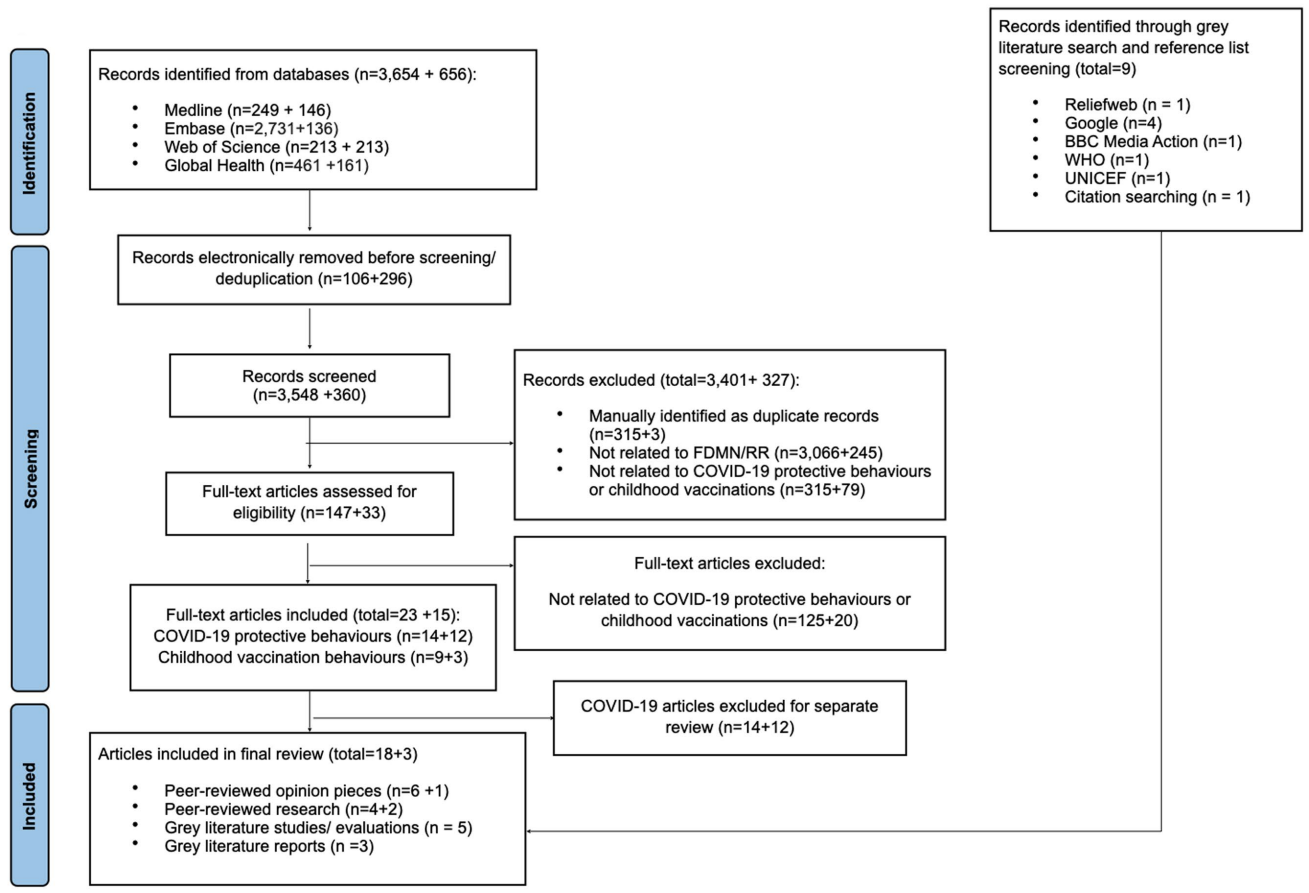
PRISMA flowchart of included studies (18).

The results shown here comprise of the original search in 2021 and the update search in 2024, Numbers are given as totals with the breakdown of each search in brackets. The systematic search identified a total of 4,310 (3,654 in 2021 + 656 in 2024) entries. After de-duplication, title/abstract screening of 3,908 (3,548 in 2021 + 360 in 2024) and full-text screening of 180 (147 in 2021 + 33 in 2024) records, 12 (9 in 2021 + 3 in 2024) articles were found to be eligible. Nine additional articles were added through grey literature searches and citation screening, leading to a total of 21 included articles (see Figure 2). Articles were excluded as not aligned with the target population, scoping review concepts or context (as per PCC see Supplementary material 2).

An overview of all included articles is provided in Tables 2, 3 (for further details see Supplementary material 5). The research landscape consists of heterogenous article types. Thirteen articles were published in peer-reviewed journals of which seven were opinion pieces (4, 30–35) and six were research articles (16, 36–40). Eight grey literature articles were found, comprised of three reports (13, 41, 42), three NGO/UN response evaluation reports (11, 43, 44) and two non-peer reviewed research studies (45, 46). For peer-reviewed and grey literature based on research, qualitative (16, 36, 40, 45) approaches and mixed methods were predominantly applied (11, 43, 44, 46), followed by quantitative (cross-sectional) (37–39) approaches. All articles were published between 2018 and 2023. Most interventions were reported from 2017 and 2018 (4, 11, 13, 30–34, 36–39, 42, 43), and one from 2020 (41).

**Table 2:** Characteristics of included peer-reviewed literature.

First author, year	Evidence source		Type of research			Target group			Location		Target behaviour		Critical appraisal (quality criteria met/total)
	Primary research	Opinion piece	Quantitative	Qualitative	Mixed methods	FDMN/Rohingya refugees	HSP	Both	Cox’s Bazar – general	Specific camps	Receiving	Facilitating	
Ahmed, 2023 (16)													7/7
Chan, 2018 (30)													6/6
Feldstein, 2020 (38)													6/7
Jalloh, 2019 (36)													7/7
Jalloh, 2020 (31)													5/6
Hsan, 2019 (33)													5/6
Hsan, 2020 (32)													5/6
Khan, 2019 (37)													6/7
Khan, 2023 (35)													4/5
Qadri, 2018 (34)													5/6
Qayum, 2023 (40)													7/7
Rahman, 2019 (4)													5/6
Summers, 2018 (39)													6/7

Grey: Applies; White: Does not Apply.

**Table 3:** Characteristics of included grey literature.

First author, year	Evidence source				NGO/UN		Target group			Location		Target behaviour		Critical appraisal (quality criteria met/total)
	Primary research	Report	Response evaluation report	Situation report	NGO	UN agency	FDMN/Rohingya refugees	HSP	Both	Cox’s Bazar – general	Specific camps	Receiving	Facilitating	
Bangladesh Health Watch 2019 (42)														22/29
BBC Media Action, Translators without Borders, 2019 (45)														16/24
BRAC, 2019 (46)														28/30
Red R India, 2018 (43)														22/30
UNHCR, 2018 (11)														28/30
UNICEF, 2018 (44)														28/30
UNICEF, 2020 (41)														15/23
SEARO WHO, 2019 (13)														22/29

Grey: Applies; White: Does not Apply.

Most articles included barriers and drivers from the perspectives of FDMN/Rohingya refugee population (4, 16, 31–33, 35–40, 45), or both HSP and FDMN/Rohingya refugees’ perspectives (11, 34, 41–44, 46). Only two articles related to HSP views only (13, 30). Information on location in the camps and population subgroups was collected. However, it was not possible to organise findings by these characteristics due to a lack of detail and heterogeneity of studied outcomes. Only one evaluation study on vaccination specific interventions was found (40), the impact for most interventions was described through authors’ reflections, post vaccination campaign coverage surveys or general evaluations of the NGO/UN response.

Critical appraisal found that included articles met a moderate to high range (between 65 and 100%) of quality criteria. On average, 80% of quality criteria were met for grey literature appraised with the AACODS, 86% for opinion pieces appraised with the JBI checklist and 90% for research studies appraised with the MMAT. Most research studies lost scores on the risk of non-response bias, whilst grey literature lost scores for lack of peer-review and editing by reputable authority (see Supplementary material 6).

### 3.2 Main barriers and drivers identified

#### 3.2.1 Individual factors

As demonstrated in Tables 4, 5, barriers and drivers relating to the *capability factor* of the COM-B model were only identified for FDMN/Rohingya refugees receiving vaccination (eight barriers and drivers reported by nine articles) and were mostly related to knowledge. No articles reported on capability barriers or drivers to HSPs facilitating vaccinations.

**Table 4:** Overview of barriers (−) and drivers (+) to receiving vaccinations (FDMN/Rohingya refugees) arranged by COM-factors.

Individual factors		Context factors	
Capability	Motivation	Social opportunity	Physical opportunity
Barriers and drivers (N = 8), articles (N = 9)	Barriers and drivers (N = 19), articles (N = 11)	Barriers and drivers (N = 7), articles (N = 8)	Barriers and drivers (N = 19), articles (N = 11)
**Knowledge** + Knowledge of why childhood vaccination is important (36, 40, 41, 43, 45), in registered camps only (16) + Parental/Father’s education (16) + Knowledge about own children’s vaccination status (46) – Lack of knowledge about vaccination campaigns in camps (37, 38), for makeshift more than registered camps (40) – Lack of knowledge about vaccine preventable diseases, vaccinations (16, 37), vaccination schedule (41) or need to get multiple vaccinations on the same day (36) – Spread of misinformation via face-to-face and electronic communication with FDMN/Rohingya refugees in camps, hosts and home county (45) **Ability** – Sickness so cannot bring children for vaccination (37) or receive vaccination (40) – In makeshift camps, forgetting to attend (40)	**Confidence/trust** + Confidence from not seeing/hearing of children with serious side effects or dying after vaccination campaigns (36) + Confidence in receiving good care from vaccination campaign staff (36) +Trust in religious leaders, elders, village doctors, pharmacists, mothers trained by NGOs as sources of information (36) + Increased trust of SP from previous vaccination campaigns (42) – Lack of trust in appointed community leaders “mahjees” on health issues due to their liaison role with the army (36) – Feeling misled by some religious leaders due to their initial instructions to refuse vaccination (36) or their practices not grounded in Qur’an (36) – Lack of trust in vaccinators’ skills and treatment of children (36) – Lack of trust in volunteers reassurance about not becoming Christian after vaccination (45) – Being denied access to healthcare in home country (4, 32, 33) **Values/beliefs** + Belief that vaccines prevent disease (36) – Belief that combination vaccinations are designed to kill the Rohingya population (36) – Beliefs that vaccination will leave mark forbidden in Islam and people will become a Christian if vaccinated (36, 45) – Competing priorities, e.g., poor health, drinking water, relief collection of adult (36, 37) and of child (38) – Do not believe that vaccines are important (40) **Emotions/impulses/feelings** + Having previously lost at least one child (16) + History of previous admission (16) – Fear of vaccine (40, 42) or multiple vaccines (36), side effects (4, 36, 37, 40, 45) including weakness, death (36, 45) and needles (38) – Fear of contracting COVID-19 in vaccination facility (41) – Fear of pain for child (37) and fear amongst children when see other children in pain who receive vaccinations (36) – Dislike taste of vaccine (in registered more than makeshift camps (40)	**Social and cultural norms** – Women and girls cannot interact or show part of their body to men outside their family (31, 36) – Norm in Myanmar for children to receive one vaccination (not combination vaccinations) (36, 37) – Norm to seek healthcare outside of formal healthcare system (39, 46) **Social Support** + Use of female vaccinators, who encourages vaccinations for women and girls and provides opportunity to ask questions (13) + Ability to ask questions to HSP and inform Camp in Charge if concerns arise (45) + Having more than two children (16) – Recent arrival of refugees – registered vs. makeshift camps (16)	**Availability of information** + Receiving information about immunization via Friday prayers, household visit, community meetings, information centres, megaphones, video documentary (13, 16, 36, 44) + Presence of electronic device (16) – Lack of information from vaccinators about vaccines and side effects (36), on why cholera vaccination is needed (37) and on time and place of vaccination campaigns (37) – Language barriers between service providers and caregivers (36, 37) **Geographical access** + Preference for vaccination sites to be in close proximity (43, 46) – Vaccination sites located too far away (37) – First access to vaccines upon arrival in camps (46) **Convenience, appeal, and appropriateness of vaccination** – Long queues at vaccination sites (37) and waiting times (40) – Short campaign durations (37) – No privacy or gender sensitivity in place at vaccination sites (31, 36) leading to preference amongst women for household visits (36) – Absence from camp during vaccination campaign–makeshift and registered camps (40) – For makeshift camps-not having time or being too busy (40) **Availability of resources** – Insufficient vaccines at vaccination sites (11, 37) – Insufficient vaccinators (36, 37) and predominantly male (36) + Father’s employment (16) – Vaccinator refusing to vaccinate (40) – Clinic was closed (40) **Rights/regulation/legislation** – Vaccination services suspended due to COVID-19 pandemic (41) – Restrictions on movement during COVID-19 pandemic (41)

**Table 5:** Overview of barriers (−) and drivers (+) to *facilitating* vaccinations (HSP) arranged by COM-factors.

Individual factors		Context factors	
Capability	Motivation	Social opportunity	Physical
Barriers and drivers (N = 0), articles (N = 0)	Barriers and drivers (N = 3), articles (N = 3)	Barriers and drivers (N = 6), articles (N = 4)	Barriers and drivers (N = 13), articles (N = 12)
	**Emotions/impulses/feelings** – Campaign fatigue with multiple dose vaccinations and continuous influx (38) **Confidence/trust** + Confidence amongst religious leaders after several months of vaccination campaigns (36) – Difficulties in convincing parents to bring children for vaccination in MSs and spontaneous settlements (13)	**Social, cultural norms and values** + Female vaccinators have easier access to families and teenage girls (13) – Socio-cultural issues (38) – Disapproval amongst religious leaders of women and girls being publicly vaccinated by men (36) **Social support** + Collaborative and efficient partnerships with other organizations (13) + Good relationship with government (13, 44) – Lack of community engagement (44)	**Availability of resources** + WHO/GAVI deliver OCV vaccinations free of charge (13) – Lack of evidence of Rohingya children’s vaccination status (36, 37) – Problems with vaccination record management (30) – Challenges establishing fully functioning health facilities (44) – Vaccination services suspended due to COVID-19 pandemic (41) – Insufficient vaccines at vaccination sites (11, 32, 34, 38) – Insufficient vaccinators (36) **Geographical access** – Scattered settlements over a large area (37) – Constant new arrivals during vaccination campaign (34, 37–39) and population movement (13, 30, 37) – Rapidly evolving conflict situation (38) **Convenience** – Inconvenience of administering multiple vaccinations compared to one (32) and intramuscular vaccinations compared to oral vaccinations (37, 38) **Rights/regulation/legislation** – Rohingya are unregistered (30, 39) – Political and resource constraints (38)

The main capability driver was refugees’ knowledge of why childhood vaccinations are important (16, 36, 40, 41, 43, 45, 46). Barriers included a lack of knowledge about how to access vaccinations such as details on the time and place of campaigns (37, 38, 40), poor awareness of the available vaccines (16, 37) and vaccination schedules (36, 41), and vaccination misinformation (45). Other barriers, each mentioned by one article, were sickness causing inability to bring children for vaccination (37) and forgetting to attend (40).

*Motivation-related factors* were also predominantly found for caregivers’ receiving vaccinations (19 barriers and drivers reported by 11 articles) rather than HSPs facilitating vaccinations (3 barriers and drivers reported by 3 articles). They could be subcategorised into motivation related factors linked to confidence/trust, values/beliefs, and emotions/impulses or feelings.

Emotion, impulse and feeling related barriers were mostly found for FDMN/Rohingya refugees receiving vaccinations. These included fear of vaccines (40, 42), pain (36, 37), side effects (4, 36, 37, 40, 45), needles (38), weakness and death (36, 45). Fear of contracting COVID-19 from vaccination facilities was also mentioned in one article (41). Drivers included previous experiences of having lost a child or previous admission to hospital (16). For HSP facilitating vaccinations, one article mentioned ‘campaign fatigue’ for multidose vaccines, when too many campaigns were undertaken (38).

Various values and beliefs around vaccinations were identified. Barriers for FDMN/Rohingya refugees included beliefs that vaccines cause people to become Christian, vaccines leave marks forbidden in Islam that prevent the vaccinated individual from going to heaven (36, 45), or that vaccines were designed to kill the Rohingya population (36). Both supportive and opposing beliefs about the importance of vaccination were reported in terms of their impact on preventing disease. Some believed that vaccines were not important (40), whilst others were motivated by the belief that vaccines effectively prevent disease (36). Three articles discussed competing priorities for FDMN/Rohingya refugees, e.g., around poor health, collection of drinking water, relief collection and attending school (36–38).

Confidence and trust were described as important factors with conflicting data regarding the role of community/religious leaders and vaccinators, being both a driver and a barrier to receiving vaccinations. A lack of trust in appointed leaders “mahjees” was noted due to their liaison role in the army and in religious leaders when they had initially instructed to refuse vaccinations, or claimed vaccination practices were not grounded in the Qur’an (36). However, trust in vaccinations was also noted when community leaders, elders, village doctors, and in particular religious leaders recommended vaccinations, following training by NGOs (36). Evidence of both confidence in receiving good care from vaccinators (36), as well as problems with vaccinators or volunteers were found. These encompassed a lack of trust in vaccinators or volunteers’ treatment of children (36), and their attempts to reassure FDMN/Rohingya refugees that they would not become Christian following a vaccination (45).

A further barrier included a lack of trust due to having been denied access to health care in their home country (4, 32, 33). Encouragingly, for caregivers and religious leaders, trust in vaccines appeared to be correlated with time and experience in Cox`s Bazar, having increased confidence in vaccines from not seeing or hearing of children having serious side effects following vaccination campaigns (36, 42). Encouragingly, for caregivers and religious leaders, trust in vaccines appeared to be correlated with time and experience in Cox`s Bazar, having increased confidence in vaccines from not seeing or hearing of children having serious side effects following vaccination campaigns (36, 42). Corresponding to the aforementioned lack of trust by FDMN/Rohingya refugees, a barrier for HSPs facilitating vaccinations discussed by one article was the difficulty in convincing parents to bring children for vaccination in makeshift and spontaneous settlements (13), whilst a driver for HSPs was the increased confidence amongst religious leaders after several months of vaccination campaigns (36).

#### 3.2.2 Context factors

Barriers and drivers related to physical and social opportunity (context factors) were identified for both caregivers and HSPs. Physical opportunity barriers and drivers were most reported for caregivers receiving vaccinations (19 barriers and drivers reported by 11 articles) and for HSPs facilitating vaccinations (13 barriers and drivers reported by 12 articles). In contrast, fewer social opportunity barriers and drivers were reported for caregivers receiving vaccinations (seven barriers and drivers across eight articles) and for HSPs facilitating vaccinations (six barriers and drivers across four articles).

*Physical opportunity related* barriers and drivers could be categorised into several key areas: the availability of information, geographical access, convenience/appeal of vaccination, availability of resources, and rights/regulation and legislation.

A common barrier and driver for receiving vaccinations for FDMN/Rohingya refugees was the availability (or lack) of information. Issues included a lack of information about the vaccines from vaccinators, the vaccination campaigns/services being held (36, 37) and language barriers (36, 37). On the other hand, information provided via different communication/awareness campaigns was a driver to increase vaccination uptake for FDMN/Rohingya refugees receiving vaccinations (13, 16, 36, 44). One article commented on the presence of an electronic device in the household being associated with higher vaccination rates (16).

The availability of resources was another significant barrier for both caregivers and HSP. Barriers involved the (in) availability of vaccines (11, 32, 34, 37, 38) and a lack of vaccinators (36, 37), which were predominantly male when available (36). For HSP specifically, further barriers such as the lack of, or problems with, vaccination records (30, 36, 37) and difficulties establishing health facilities (44) were identified.

Geographical access, logistical and appeal issues also played a crucial role for caregivers receiving vaccinations and HSPs facilitating vaccinations. The scattered settlements made accessing and delivering vaccinations difficult (37, 43, 46), with a preference for vaccination sites to be in close proximity (43, 46). In addition, the influx and constant movement of refugees made facilitating vaccinations for HSP challenging (13, 30, 34, 37–40). Logistical issues for HSPs included the relatively complex administration of multidose and/or intramuscular vaccinations (32, 37, 38) in comparison to oral vaccines (37). For FMDN/Rohingya refugees, long queues (37), short campaign durations (37), and vaccination sites with limited privacy or gender considerations (31, 36), and insufficient female vaccinators, reduced the appeal of vaccination facilities (31, 36). This led to FDMN/Rohingya refugee women preferring to receive vaccinations during household visits (36).

Rights, regulation and legislation related barriers were also found for both caregivers and HSP, though these were less frequently mentioned. Amongst these were restriction of movements and vaccination activities during the COVID-19 pandemic (41), lack of registration (30, 39), and political and resource constraints (38).

*Social opportunity* barriers and drivers could be subcategorised to social and culture-related norms, and social support.

Socio-cultural barriers were linked to healthcare and gender norms. These related to norms in Myanmar to seek healthcare outside the formal healthcare system (39, 46), or it being common to receive one single vaccination a day, instead of multiple or combination vaccines offered in Cox’s Bazar (36, 37). Gender norm barriers were reported for receiving and facilitating vaccination in three articles (13, 31, 36), corresponding with the aforementioned physical opportunity barrier of having predominantly male vaccinators. These included women and girls not being able to interact or show parts of their body to men outside their family (31, 36), and religious leaders not recommending vaccinations as they disapproved of women and girls being publicly vaccinated by men (36). Accordingly, female vaccinators were described as drivers to facilitate vaccinations for HSP as they had easier access to families and teenage girls (13).

Social support was shown to be a driver for both refugees receiving as well as for HSPs facilitating vaccinations. For FDMN/Rohingya refugees, being able to ask questions to NGO or camp staff (45)—particularly through the use of female vaccinators who could encourage and enable women and girls to speak up—was identified as a driver (13). In contrast, the recent arrival of refugees in registered and makeshift camps was seen as a barrier (16). For HSP, collaborative partnerships and good relationships with the government (13, 44) were identified as drivers. Barriers here included a lack of community engagement to facilitating vaccinations (44).

#### 3.2.3 Assessing differences

All four COM factors were relevant to FDMN/Rohingya refugees, while three COM factors (excluding capability) applied to HSP. For refugees receiving vaccinations, motivation and physical opportunity related barriers and drivers were the most identified, whereas for HSPs facilitating vaccinations, physical opportunity related barriers and drivers were most reported.

Some authors investigated differences between makeshift and registered camps (16, 38, 40), registered and unregistered refugees (39) or different lengths of stay (37), though it was difficult to draw clear conclusions. Lower vaccination rates were generally found in recently arrived, unregistered FDMNs or those living in makeshift camps in comparison to those who had lived there since before 2017 in registered camps (16, 38, 39), explained by registered refugees being more aware of local practices and improved access to health facilities (38, 39). However, Khan et al. found relatively higher coverage of the oral cholera vaccine among people living in camps for 4–6 months compared to people living there for more than 6 months, but no association between vaccination coverage and length of stay for all other vaccines (37). Although we did not look specifically focus on the host community, it is noteworthy that Qayum et al. highlighted higher oral cholera vaccine coverage in the registered and makeshifts camps in comparison to the host community (40). This was attributed to the host community being unaware of the vaccination campaigns, unavailability of vaccines, and unawareness of where vaccines were available (40). Ahmed et al. also investigated other factors associated with good vaccination practices and found higher vaccination rates among families where fathers were employed and where parents had a higher level of education (16).

### 3.3 Interventions related to childhood vaccinations

Interventions to remove barriers and strengthen drivers to receiving and facilitating childhood vaccination are shown in Table 6. Implemented interventions were identified in 20 articles, described as part of authors’ reflections (4, 30, 32–34), post-campaign vaccination coverage surveys (31, 35, 37–39), reports (13, 41, 42), NGO/ UN response evaluation reports (11, 43, 44, 46) and research studies (16, 36). Just one evaluation study of a vaccination campaign was found (40). Generally, descriptions of interventions were brief and lacked detail, which made separate charting of interventions for different populations and their target behaviours impossible.

**Table 6:** Implemented interventions (with and without evaluation) charted by COM-factor and intervention type from the BCW.

Intervention type*	COM-factor addressed*	Intervention
Information/education	Capability Motivation	Dissemination of vaccination messages in community settings: Friday prayers and faith-based messaging, household visits (42), residential community meetings, health centres (31, 36), narrowcasting (13) and community theatre (13) Dissemination of vaccination messages in video-documentary format (36) Reminders (31) and location (36) on the day of vaccination campaign announced by community mobilisers through loudspeakers (“miking”) and bullhorns (44) Radio campaigns focused on when/where vaccination is available vaccination importance (13) and on caregiver vaccination concerns (31) Trusted model mothers identified and engaged to answer vaccination questions (31) Undertaking focus group discussions to understand community needs and improve target messages (13, 31, 44) Additional health education on WASH measures to prevent infection (35)
Persuasion	Motivation	Local volunteers, community health workers, religious leaders and mahjees enlisted to promote vaccination campaigns (31, 34, 42)
Incentivisation	Motivation	/
Coercion	Motivation	/
Training	Capability Physical opportunity Motivation	Training of model mothers to answer questions about vaccination concerns (31) Using skilled professionals for vaccination (13, 37)
Restriction	Physical & social opportunity	/
Environmental restructuring	Physical & social opportunity Motivation	Supplemental immunisation activities including time-limited, fixed-site vaccination campaigns (4, 11, 16, 30, 32–34, 37–41, 44, 46), with community outreach by healthcare facilities with mobile teams (13, 39, 42) Establishing EPI program (13, 16, 37, 41, 43, 44, 46) as part of a two-pronged approach (13, 44) ‘Sweep’ activity for vaccinating missed cases through identification of Rapid Convenience Monitoring (42) Co-ordinated, inter-sectoral partnerships (13, 34, 37, 39, 42, 44) with long-term community engagement (36) Social mobilisation/community mobilisers (including volunteers and community health workers) (13, 16, 35, 41, 42, 44) to identify eligible children (31, 36), for home visits for vaccination promotion (42), administration and follow-up (4, 42) Yellow or Moni flags to indicate vaccination site (35, 36) Home visits (36), provision of private areas for women and adolescent girls to be vaccinated (31), and increasing number of female vaccinators (31) COVID-19 infection prevention control measures at health facilities (41) Opportunistic vaccination of contacts of diphtheria cases in home/health facility (4) and contact tracing (4, 13) Oral (37) and single-dose schedule vaccination administration where safe and effective (30, 32, 34) Distribution of soap for each beneficiary (35) Extensive WASH interventions to support vaccination efforts for cholera (35)
Modelling	Social opportunity Motivation	Engaging model mothers and female hafiz (31, 36) Local volunteers, community health workers, religious leaders and mahjees enlisted to promote vaccination campaigns (31, 34, 42)

*Based on TIP handbook and Michie et al. The behaviour change wheel: A new method for characterising and designing behaviour change interventions (18, 24).

Intervention types as per the BCW included mostly environmental restructuring interventions (12 interventions reported by 20 articles), and most frequently involved supplemental (4, 11, 16, 30, 32–34, 37–41, 44, 46) and routine immunisation campaigns (13, 16, 37, 41, 43, 44, 46). Other types of interventions were information/education (seven interventions reported by six articles) (13, 31, 35, 36, 42, 44), modelling (two interventions reported by four articles) (31, 34, 36, 42), with least interventions in the areas of persuasion (one intervention reported by three articles) (31, 34, 42) and training (two interventions reported by three articles) (13, 31, 37). No interventions relating to incentivisation, coercion and restriction were identified. Combining interventions for vaccinations with other measures (e.g., WASH, health education) and intersectoral partnerships were also reported and deemed successful or important (13, 34–37, 39, 42, 44).

We also reviewed the author’s recommendations for possible future interventions. Five articles recommended five interventions (30, 32, 36, 40, 46) for which from the included literature no evidence of their implementation was found. These recommendations were rather broad and included information/education type interventions such as investigating interactions between HSP and caregivers (36), exploring alternative vaccination schedules (46), and providing vaccination cards and medical summaries (30). Correlating with previously mentioned drivers to vaccination uptake, it was recommended to work closely with religious leaders to identify appropriate passages from the Qur’an and Hadith which can be used to support vaccination uptake (31), or encourage the presence of the camp administration during vaccination campaigns to improve efficiency (40). Given the dynamic and evolving conflict situation, some recommendations from older articles were subsequently implemented and reported in more recent articles (see Table 6).

## 4 Discussion

We reviewed 21 articles that explored barriers, drivers and interventions regarding childhood vaccination behaviours in Cox’s Bazar, guided by the COM-B framework (18) and BCW (24). Only six articles were peer-reviewed primary research, with remaining articles being opinion pieces, grey literature reports/studies and response evaluations. Evidence was available for barriers and drivers to FDMN/Rohingya refugees receiving and HSP facilitating vaccinations. The barriers and drivers were mostly linked to the COM factors of physical opportunity, followed by motivation. Most frequently reported implemented interventions focused on environmental restructuring and information/education. Only one evaluation study of an intervention was found. Below we discuss the scope of, and gaps in the research landscape followed by a brief discussion of key barriers and their implications for interventions.

### 4.1 Barriers and drivers to childhood vaccination behaviours

The review identified a range of barriers and drivers on both the demand (FDMN/Rohingya refugee) and supply (HSP) side, though more evidence for barriers and drivers for FDMN/Rohingya refugees was found than for HSPs. Barriers and drivers for all four COM factors was described for FDMN/Rohingya refugees receiving vaccinations, whilst for HSPs facilitating vaccinations, three COM factors were found with no articles reporting capability barriers or drivers. Both motivation factors (e.g., fears, trust) and physical opportunity factors (e.g., availability of vaccines) were most reported for FDMN/Rohingya refugees receiving vaccinations, whilst physical opportunity factors (e.g., geographical access, availability of resources) were most reported for HSPs facilitating vaccinations. The less frequent reporting of barriers and drivers for HSPs highlights a need for further exploration of their perspectives in the vaccination efforts, given the well-established and critical role of HSPs in improving vaccination coverage (47–49).

The multiple individual and context influences on childhood vaccination behaviours identified in this review align closely with those reported in the WHO ‘Global Evidence Review on Health and Migration’ (19). Wide-ranging environmental (physical opportunity) barriers for caregivers and HSPs alike are expected in a challenging environment such as Cox’s Bazar, where a lack of infrastructure and resources are an obvious and immediate barrier to service delivery (19, 50). Indeed many of the environmental barriers reflect the six building blocks of the WHO health systems Building Blocks framework (51): (i) service delivery; (ii) health workforce; (iii) information; (iv) medical products, vaccines and technologies; (v) financing; and (vi) leadership and governance. Additionally, motivation related barriers identified may be linked to a lack of vaccine confidence, which is known to be influenced by political and medical mistrust (52–54). For FDMN/Rohingya refugees in particular, this may be associated with persecution and violence experienced in their home country (13, 44). Additionally for many caregivers, Cox’s Bazar provided first time exposure to vaccinations (38, 40, 55), particularly combination vaccines, highlighting knowledge gaps and a lack of confidence. This was further evidenced by increased vaccination rates in those that lived in registered camps and had been in the area for longer (40). Though there was some evidence of increasing trust and confidence in vaccines and vaccinators with time (36), ongoing issues with mistrust—a common phenomenon seen globally—suggest that further modes of trust building may need to be explored (56).

Social norms, social support and community/religious leader engagement have been found to be crucial for effective vaccination programs (57), though only one article explored community/religious leaders recommending vaccinations. Gender norms are likely to be important for a Muslim community where teenage girls and women practice “purdah,” the Islamic practice requiring women to be veiled from public gazes or remain within private spaces of the family (58). Understanding and addressing these leaders’ barriers and drivers may be a potential opportunity for vaccination promotion (31, 59) as they play a central role in trust building for vaccination campaigns (60).

A further evidence gap emerged with regard to different camps and subgroups of FDMN/Rohingya refugees. There was insufficient detail in most studies to identify barriers and drivers that were specific to camps or population subgroups. This is a limitation in the existing literature as treating FDMN/Rohingya refugees as a homogeneous group prevents the development of tailored and targeted interventions (20). It was notable that a few more recent studies did compare FDMN populations in registered and makeshift camps, though further research comparing barriers and drivers for different population groups and different camps would be helpful.

### 4.2 Interventions

The high number of articles describing implemented interventions with some information on impact is a strength of the literature, and the implemented interventions identified are in line with a global review of interventions to reduce VPD burden amongst migrants and refugees (61). However, the interventions lacked detail on intervention rationale, theoretical underpinning, and target populations or behaviours. Furthermore, evaluating their impact was difficult due to limited descriptions of the intervention’s impact and a lack of formal evaluation studies, and instead reliance on author’s reflections or using single indicators to measure vaccination coverage in many articles. It is therefore difficult to understand if and how these interventions work, and if they should be replicated. This lack of detailed description and evaluation is evident in the wider vaccination (62–64) and public health intervention literature (65, 66). Standardized reporting checklists for interventions (65) and increasing the publication of monitoring and evaluation reports (65, 67) would allow effective interventions to be transferred and scaled up, and increase overall transparency (62, 68, 69).

Interventions to remove barriers and strengthen drivers to childhood vaccination behaviours encompassed five intervention types (18, 24)—mainly environmental restructuring and information/education, less often modelling, persuasion and training—which means that in theory, all COM factors can be addressed (see Table 1). Environmental restructuring interventions reduce the physical and social opportunity and motivation related barriers and strengthen these drivers, whilst education interventions address capability and motivation related barriers and drivers. Overall, the interventions found correlate theoretically with the COM-factors of the most identified barriers and drivers. Those focused on improving the health system, once again align with the WHO health systems Building Blocks framework (51). No article described incentivisation, coercion or restriction interventions, which may offer additional strategies.

The limited available intervention impact data suggests that vaccination campaigns, routine immunisation services, community mobilisation and gender specific interventions may improve vaccination uptake (4, 11, 13, 31, 32, 36, 37, 39, 41–44, 46), aligning with findings of a systematic review (19, 63). Tailored environmental restructuring interventions, such as the provision of female vaccinators and private vaccination areas, were found to address gender specific barriers to vaccination uptake (13, 31), and strengthening these would be in line with the Global Immunization Agenda 2030 which includes gender equity as a strategic priority (70). In addition, collaborating with community leaders may offer alternative, culturally appropriate intervention strategies (31, 71).

### 4.3 Research landscape

Regarding the research landscape, encouragingly, critical appraisal found the articles themselves to be of moderate to high quality. However, the literature and data were frequently scattered, and difficult to collate as embedded in different parts of articles and described in varying detail. This is in keeping with the known difficulty in humanitarian conflict settings to collate and share information systematically, due to logistical challenges, problematic data collection and a lack of health information sharing mechanisms (72, 73).

### 4.4 Strengths and limitations

To our knowledge, this is the first scoping review on childhood vaccinations in the Rohingya population. Its strengths include the comprehensive search strategy applying the JBI methodology, and a theory-informed analysis utilising the COM-B model and BCW that are useful in understanding and addressing public health challenges (18, 24). These behaviour frameworks have been extensively applied to childhood and adult vaccination behaviours (74–76) and provide the theoretical underpinning of the WHO Tailoring Immunization Programmes (18). Their use enabled a holistic and systematic examination of individual and context barriers and drivers reported in the literature, avoiding “blind spots” (20), and classification of different types of reported interventions into a well-accepted framework (BCW). In addition, given the scattered evidence base, this review adds value by providing and overarching overview of barriers and drivers. Furthermore, this review provides a nuanced examination of vaccination behaviours related to receiving and facilitating vaccinations, thereby reviewing demand and supply side influences on vaccination coverage and equity outcomes.

However, several limitations should be acknowledged. Despite an extensive search strategy and screening four databases, reference lists, and grey literature sources, further literature may exist. We used the Google Advanced Search function which has a low reproducibility, but it was useful as we found reports which were not displayed on the respective organisation’s websites. This emphasises again the need for improved mechanisms to share and collate evidence. The results may also be affected by publication bias. As mentioned before, especially in conflict settings, frontline organisations and researchers may not have the capacity to publish findings (73). Additionally, even though no language restrictions were set, we only used English search terms and only found English articles. Studies exploring barriers and drivers to health behaviours typically use self-reported accounts and this was evident for most of the included articles. We were unable to clearly differentiate barriers and drivers by the specific type of vaccine or disease such as measles due to limited detail of the data, and it would be useful for future research to investigate this. It is important to recognise that frequency counts were undertaken to describe the range of evidence and identify most reported barriers or drivers. However, frequency of reporting may not correlate with the impact of this barrier or driver, and configuration of the data as a whole is equally important.

A limitation of the BCW is the wide range of interventions captured in the intervention type “environmental restructuring” potentially resulting in vague guidance for appropriate interventions to change the social or physical context. This was sufficient for classifying interventions in our review. However, researchers could usefully employ more specific behaviour change techniques or mechanisms of action (77) to improve the links between barriers and interventions.

## 5 Conclusion

A wide range of barriers and drivers for FDMN/Rohingya refugees receiving and HSPs facilitating vaccinations in Cox’s Bazar exist, with more barriers and drivers reported from FDMN/Rohingya refugees perspective compared to HSPs perspective. Context and motivation related factors were the most frequently identified barriers and drivers. Salient gender and social norms also played a significant role in influencing both behaviours. However, available data was insufficiently described to assess geographical or subgroup differences within Cox’s Bazar. Encouragingly, several interventions were reported that address these barriers and drivers, though reported interventions lacked detail and the description of their impact and evaluation were very limited. Community and faith leaders play an influential role in Rohingya culture and could be key partners in strengthening vaccination programs and community mobilisation efforts in the future. Overall, this review emphasised the need for further research, particularly on HSP perspectives, subgroups within the Rohingya population, and better reporting, monitoring, and evaluation of interventions to design targeted strategies for increasing vaccination uptake in the Rohingya community in Cox’s Bazar.

## Data Availability

The original contributions presented in the study are included in the article/Supplementary material, further inquiries can be directed to the corresponding author.

## Footnotes

^1^ https://osf.io/n6d3z
