# Supplementary material for: Barriers and drivers to childhood vaccinations in Forcibly Displaced Myanmar Nationals (FDMN)/Rohingya refugees in Cox’s Bazar, Bangladesh: a scoping review
Source: Front Public Health. 2025 Jul 18;13:1592452. doi: 10.3389/fpubh.2025.1592452 (PMC12313643; doi:10.3389/fpubh.2025.1592452)
Supplement: Supplementary file 1 [file Data_Sheet_1.pdf]

# Barriers and drivers to childhood vaccinations in Forcibly Displaced Myanmar Nationals (FDMN)/ Rohingya refugees in Cox's Bazar, Bangladesh: A scoping review

## Supplements

Supplement 1: Preferred Reporting Items for Systematic reviews and Meta-Analyses extension for Scoping Reviews Checklist (PRISMAScR)........ 2
Supplement 2. Eligibility Criteria based on Population-Concept-Context (PCC) Framework ........ 4
Supplement 3: Search strategy syntaxes ........ 5
Supplement 3.1: Search strategy syntax for included databases (COVID-19 and childhood vaccination search strategy) ........ 5
Supplement 3.2: Search strategy syntax for update search ........ 7
Supplement 4: Information sources and search strategy of grey literature search ........ 9
Supplement 5: Overview of included literature ........ 10
Supplement 5.1 Overview of peer-reviewed literature ........ 10
Supplement 5.2 Overview of grey literature ........ 13
Supplement 6: Critical appraisal of included articles ........ 15
Supplements reference list ........ 20

**Supplement 1: Preferred Reporting Items for Systematic reviews and Meta-Analyses extension for Scoping Reviews Checklist (PRISMAScR)**

| SECTION | ITEM | PRISMA-ScR CHECKLIST ITEM | REPORTED IN |
|---|---|---|---|
| **TITLE** | | | |
| Title | 1 | Identify the report as a scoping review. | Title |
| **ABSTRACT** | | | |
| Structured summary | 2 | Provide a structured summary that includes (as applicable): background, objectives, eligibility criteria, sources of evidence, charting methods, results, and conclusions that relate to the review questions and objectives. | Abstract |
| **INTRODUCTION** | | | |
| Rationale | 3 | Describe the rationale for the review in the context of what is already known. Explain why the review questions/objectives lend themselves to a scoping review approach. | Introduction |
| Objectives | 4 | Provide an explicit statement of the questions and objectives being addressed with reference to their key elements (e.g., population or participants, concepts, and context) or other relevant key elements used to conceptualize the review questions and/or objectives. | Introduction |
| **METHODS** | | | |
| Protocol and registration | 5 | Indicate whether a review protocol exists; state if and where it can be accessed (e.g., a Web address); and if available, provide registration information, including the registration number. | Methods |
| Eligibility criteria | 6 | Specify characteristics of the sources of evidence used as eligibility criteria (e.g., years considered, language, and publication status), and provide a rationale. | Methods, Supplement |
| Information sources | 7 | Describe all information sources in the search (e.g., databases with dates of coverage and contact with authors to identify additional sources), as well as the date the most recent search was executed. | Methods, Supplement |
| Search | 8 | Present the full electronic search strategy for at least 1 database, including any limits used, such that it could be repeated. | Supplement |
| Selection of sources of evidence† | 9 | State the process for selecting sources of evidence (i.e., screening and eligibility) included in the scoping review. | Methods |
| Data charting process | 10 | Describe the methods of charting data from the included sources of evidence (e.g., calibrated forms or forms that have been tested by the team before their use, and whether data charting was done independently or in duplicate) and any processes for obtaining and confirming data from investigators. | Methods |
| Data items | 11 | List and define all variables for which data were sought and any assumptions and simplifications made. | Methods Supplement |
| Critical appraisal of individual sources of evidence§ | 12 | If done, provide a rationale for conducting a critical appraisal of included sources of evidence; describe the methods used and how this information was used in any data synthesis (if appropriate). | Methods |
| Synthesis of results | 13 | Describe the methods of handling and summarizing the data that were charted. | Methods |
| **RESULTS** | | | |
| Selection of sources of evidence | 14 | Give numbers of sources of evidence screened, assessed for eligibility, and included in the review, with reasons for exclusions at each stage, ideally using a flow diagram. | Results, Figure 2 |
| Characteristics of sources of evidence | 15 | For each source of evidence, present characteristics for which data were charted and provide the citations. | Tables 2.1, 2.2, 3.1, 3.2, Supplement |
| Critical appraisal within sources of evidence | 16 | If done, present data on critical appraisal of included sources of evidence (see item 12). | Supplement |
| Results of individual sources of evidence | 17 | For each included source of evidence, present the relevant data that were charted that relate to the review questions and objectives. | Tables 2.1, 2.2, 3.1, 3.2, Supplement |
| Synthesis of results | 18 | Summarize and/or present the charting results as they relate to the review questions and objectives. | Results, Tables 2.1, 2.2, 3.1, 3.2, |

| SECTION | ITEM | PRISMA-ScR CHECKLIST ITEM | REPORTED IN |
|---|---|---|---|
| **DISCUSSION** | | | |
| Summary of evidence | 19 | Summarize the main results (including an overview of concepts, themes, and types of evidence available), link to the review questions and objectives, and consider the relevance to key groups. | Discussion |
| Limitations | 20 | Discuss the limitations of the scoping review process. | Discussion |
| Conclusions | 21 | Provide a general interpretation of the results with respect to the review questions and objectives, as well as potential implications and/or next steps. | Discussion |
| **FUNDING** | | | |
| Funding | 22 | Describe sources of funding for the included sources of evidence, as well as sources of funding for the scoping review. Describe the role of the funders of the scoping review. | Funding |

*From:* Tricco AC, Lillie E, Zarin W, O'Brien KK, Colquhoun H, Levac D, et al. PRISMA Extension for Scoping Reviews (PRISMAScR): Checklist and Explanation. Ann Intern Med. 2018;169:467–473. doi: 10.7326/M18-0850.

**Supplement 2. Eligibility Criteria based on Population-Concept-Context (PCC) Framework**

| | Including | Excluding | Justification/ Remarks |
|---|---|---|---|
| Population | FDMN/Rohingya refugees' caregivers and children<br><br>Health service providers working with FDMN/Rohingya refugees | Host communities | Vaccination uptake for host communities is high (1) |
| Concept | Barriers and drivers to receiving, administering, or recommending childhood vaccinations<br><br>Interventions to remove barriers and strengthen drivers to receiving, administering, or recommending childhood vaccination (recommended, implemented, implemented with evaluation) | Articles on VPD rates only or modelling studies<br><br>COVID-19 vaccination | |
| Context | Cox's Bazar refugee camps (general or specific), Bangladesh | Contexts outside of Cox's Bazar | |
| Article Type | Peer-reviewed articles<br>Primary and secondary research<br>Review articles<br>Text and Opinion pieces<br><br>Grey literature:<br>Summary reports by international- and non-governmental organizations<br>Research by international- and non-governmental organizations | Media/ newspaper articles, press releases, news statements, blogs<br><br>Abstracts only<br><br>Daily, weekly, or monthly situation reports or fact sheets | Media articles and press releases were excluded due lack of detail, high volume, and time limitations<br><br>Epidemiological or humanitarian situation reports that were published on daily, weekly, or monthly basis as updates were excluded as important findings are summarized in quarterly or yearly reports.<br><br>Conference abstracts or articles with abstracts only were excluded due to lack of detail.<br><br>Text and opinion pieces were deemed appropriate to include as they can hold important contextual information in humanitarian crisis settings, ("knowing how" type of evidence) (2) |
| Time Frames | No time frame | | Rohingya refugees have existed in Cox's Bazar since 1948, though major influx occurred in 2017 |
| Language | English literature | | Language barrier of researchers |

# Supplement 3: Search strategy syntaxes

Supplement 3.1: Search strategy syntax for included databases (COVID-19 and childhood vaccination search strategy)

| Database | Date of search | Search strategy syntax | | Results |
|---|---|---|---|---|
| Ovid MEDLINE | 28/08/2021 | 1 | (Bangladesh or "Cox's Bazaar" or "Cox's Bazar" or Myanmar or Rohingya or Rakhine State or Bengal).mp. | 249 |
| | | 2 | myanmar/ or bangladesh/ | |
| | | 3 | 1 or 2 | |
| | | 4 | (Rohingya or "Forcibly displaced Myanmar National" or FDMN or Refugee* or displace* or migra* or asylum seek* or undocumented or immigra* or transient*).mp. | |
| | | 5 | exp "emigrants and immigrants"/ or refugees/ or "transients and migrants"/ or Refugee camps/ | |
| | | 6 | 4 or 5 | |
| | | 7 | (Covid* or Sars-CoV-2 or coronavirus).mp. | |
| | | 8 | exp COVID-19/ or exp coronavirus/ or exp sars-cov-2/ | |
| | | 9 | 7 or 8 | |
| | | 10 | (vaccin* or immun*).mp. | |
| | | 11 | exp immunization/ or immunization schedule/ or vaccination/ or mass vaccination/ | |
| | | 12 | 10 or 11 | |
| | | 13 | 3 and 6 and (9 or 12) | |
| | | 14 | from 13 keep 1-249 | |
| GLOBAL HEALTH | 29/08/2021 | 1 | (Bangladesh or "Cox's Bazaar" or "Cox's Bazar" or Myanmar or Rohingya or "Rakhine State" or Bengal).mp. [mp=abstract, title, original title, broad terms, heading words, identifiers, cabicodes] | 461 |
| | | 2 | bangladesh/ or myanmar sh.mp. [mp=abstract, title, original title, broad terms, heading words, identifiers, cabicodes] | |
| | | 3 | 1 or 2 | |
| | | 4 | (Rohingya* or "Forcibly displaced Myanmar National" or FDMN or displace* or migra* or asylum seek* or undocumented or immigra* or transient*).mp. | |
| | | 5 | exp refugees/ or exp migrants/ or exp displacment/ | |
| | | 6 | 4 or 5 | |
| | | 7 | (Covid* or Sars-CoV-2 or Coronavirus or Corona-virus).mp. [mp=abstract, title, original title, broad terms, heading words, identifiers, cabicodes] | |
| | | 8 | exp pandemics/ or "human coronaviruses".mp. | |
| | | 9 | 7 or 8 | |
| | | 10 | (vaccin* or immun*).mp. | |
| | | 11 | vaccination/ or exp immunization/ or exp vaccines/ or (disease prevention or immunization programmes or outbreaks or epidemiology or vaccination or epidemics or immunization or campaigns or disease control or vaccines).sh. | |
| | | 12 | 10 or 11 | |
| | | 13 | 3 and 6 and (9 or 12) | |
| | | 14 | from 13 keep 1-461 | |

| | | | | |
|---|---|---|---|---|
| Embase Classic+Embase | 06/09/2021 | 1 | (Bangladesh or "Cox's Bazaar" or "Cox's Bazar" or Myanmar or Rohingya or Rakhine State or Bengal).mp. [mp=title, abstract, heading word, drug trade name, original title, device manufacturer, drug manufacturer, device trade name, keyword, floating subheading word, candidate term word] | 2,731 |
| | | 2 | bangladesh/ or Myanmar/ | |
| | | 3 | 1 or 2 | |
| | | 4 | (Rohingya or "Forcibly displaced Myanmar National" or FDMN or Refugee* or displace* or migra* or asylum seek* or undocumented or immigra* or transient*).mp. [mp=title, abstract, heading word, drug trade name, original title, device manufacturer, drug manufacturer, device trade name, keyword, floating subheading word, candidate term word] | |
| | | 5 | exp "Rohingya (people)"/ or refugee camp/ or refugee/ or undocumented immigrant/ or asylum seeker/ | |
| | | 6 | 3 or 4 | |
| | | 7 | (Covid* or Sars-CoV-2 or coronavirus or corona-virus).mp. [mp=title, abstract, heading word, drug trade name, original title, device manufacturer, drug manufacturer, device trade name, keyword, floating subheading word, candidate term word] | |
| | | 8 | coronavirus disease 2019/ or SARS-CoV-2 vaccine/ or Severe acute respiratory syndrome coronavirus 2/ | |
| | | 9 | 7 or 8 | |
| | | 10 | (vaccin* or immuni?ation).mp. [mp=title, abstract, heading word, drug trade name, original title, device manufacturer, drug manufacturer, device trade name, keyword, floating subheading word, candidate term word] | |
| | | 11 | immunization/ or vaccine hesitancy/ or vaccine/ | |
| | | 12 | 10 or 11 | |
| | | 13 | 3 and 6 and (9 or 12) | |
| Web of Science | 07/10/2021 | | Bangladesh or "Cox's Bazaar" or "Cox's Bazar" or Myanmar or Rohingya or "Rakhine State" or Bengal (Topic) and Rohingya or "Forcibly displaced Myanmar National" or fdmn or Refugee* or displace* or migra* or "asylum seek*" or undocumented or immigra* or transient* (Topic) and Covid* or "Sars-CoV-2" or coronavirus or vaccin* or immun* (Topic) | 213 |

## Supplement 3.2: Search strategy syntax for update search

| Database | Date of search | Search strategy syntax | | Results |
|---|---|---|---|---|
| Ovid MEDLINE | 06/06/2024 | 1 | Arakan or Bangladesh or "Cox's Bazaar" or "Cox's Bazar" or Myanmar or Rohingya or Rakhine State or Bengal | 146 |
| | | 2 | myanmar/ or bangladesh/ | |
| | | 3 | 1 or 2 | |
| | | 4 | Asylum or asylum seek* or displace* or diaspora or "Forcibly displaced Myanmar National" or enslaved or FDMN or "Forced migrat*" or ethni* or foreign* or "humanitarian crisis" or "humanitarian intervention*`" or illegal or immigrant* or migra* or minorit* or "People-of-concern" or "Persons-of-concern" or refugee* or Rohingya* or separated or smuggl* or traffick* or transient* or Transmigra* or Undocumented | |
| | | 5 | exp "emigrants and immigrants"/ or refugees/ or "transients and migrants"/ or Refugee camps/ or exp "Human Migration"/ or exp "Enslaved persons"/ or exp "Human trafficking” | |
| | | 6 | 4 or 5 | |
| | | 7 | vaccin* or immun* or "Immuni#ation schedule" or "vaccin* schedule" or "Immuni#ation program*" or "vaccin* program*" or "Mass vaccination" or "mass immuni#ation" or "vaccin* campaign*" or "immuni#ation campaign*" or "vaccin* refusal" or "vaccin* hesitan*" or "immuni#ation refusal" or "immuni#ation hesitancy" or "vaccin* cover*" or "immuni#ation cover*" or "vaccine-preventable-disease*" | |
| | | 8 | exp immunization/ or immunization program/ or immunization schedule/ or exp vaccination/ or exp vaccination coverage/ or mass vaccination/ or exp Vaccine-Preventable Diseases/ or exp vaccination refusal/ or exp vaccination hesitancy/ | |
| | | 9 | 7 or 8 | |
| | | 10 | 3 and 6 and 9 | |
| GLOBAL HEALTH | 13/06/2024 | 1 | Arakan or Bangladesh or Bengal or "Cox's Bazaar" or "Cox's Bazar" or Myanmar or "Rakhine State" or Rohingya | 161 |
| | | 2 | exp bangladesh/ or exp myanmar/ or "exp bay of bengal"/ or bengal.mp | |
| | | 3 | 1 or 2 | |
| | | 4 | Asylum or asylum seek* or displace* or diaspora or "Forcibly displaced Myanmar National" or enslaved or FDMN or "Forced migrat*" or ethni* or foreign* or "humanitarian crisis" or "humanitarian intervention*`" or illegal or immigrant* or migra* or minorit* or "People-of-concern" or "Persons-of-concern" or refugee* or Rohingya* or separated or smuggl* or traffick* or transient* or Transmigra* or Undocumented | |
| | | 5 | exp ethnic groups/ or exp ethnicity/ or exp emergency relief/ or exp migrants/ or exp refugees/ | |
| | | 6 | 4 or 5 | |
| | | 7 | vaccin* or immun* or "Immuni#ation schedule" or "vaccin* schedule" or "Immuni#ation program*" or "vaccin* program*" or "Mass vaccination" or "mass immuni#ation" or "vaccin* campaign*" or "immuni#ation campaign*" or "vaccin* refusal" or "vaccin* hesitan*" or "immuni#ation refusal" or "immuni#ation hesitancy" or "vaccin* cover*" or "immuni#ation cover*" or "vaccine-preventable-disease*" | |
| | | 8 | exp immunization/ or exp immunization programmes/ or exp vaccination/ or exp mandatory vaccination/ or exp oral vaccination/ or exp vaccination refusal/ or exp vaccines/ or exp mass vaccines/ | |
| | | 9 | 7 or 8 | |
| | | 10 | 3 and 6 and 9 | |

| | | | | |
|---|---|---|---|---|
| Embase Classic+Embase | 06/06/2024 | 1 | Arakan or Bangladesh or Bengal or "Cox's Bazaar" or "Cox's Bazar" or Myanmar or "Rakhine State" or Rohingya | 136 |
| | | 2 | exp bangladesh/ or exp bangladeshi/ or exp Myanmar/ | |
| | | 3 | 1 or 2 | |
| | | 4 | Asylum or asylum seek* or displace* or diaspora or "Forcibly displaced Myanmar National" or enslaved or FDMN or "Forced migrat*" or ethni* or foreign* or "humanitarian crisis" or "humanitarian intervention*`" or illegal or immigrant* or migra* or minorit* or "People-of-concern" or "Persons-of-concern" or refugee* or Rohingya* or separated or smuggl* or traffick* or transient* or Transmigra* or Undocumented | |
| | | 5 | exp asylum seeker/ or exp refugee/ or exp asylum seeker center/ or exp undocumented immigrant/ or smuggling/ or exp "Rohingya (people)"/ or exp refugee camp/ or exp refugee crisis/ or exp minority group/ or exp migration/ or exp forced migration/ or exp immigration/ or exp immigrant/ or exp ethnic group/ or exp humanitarian crisis/ or exp humanitarian intervention/ | |
| | | 6 | 4 or 5 | |
| | | 7 | vaccin* or immun* or "Immuni#ation schedule" or "vaccin* schedule" or "Immuni#ation program*" or "vaccin* program*" or "Mass vaccination" or "mass immuni#ation" or "vaccin* campaign*" or "immuni#ation campaign*" or "vaccin* refusal" or "vaccin* hesitan*" or "immuni#ation refusal" or "immuni#ation hesitancy" or "vaccin* cover*" or "immuni#ation cover*" or "vaccine-preventable-disease*" | |
| | | 8 | exp vaccination/ or exp immunization/ or exp vaccination coverage/ or exp mass immunization/ or exp vaccine hesitancy/ or exp vaccine preventable disease/ | |
| | | 9 | 7 or 8 | |
| | | 10 | 3 and 6 and 9 | |
| Web of Science | 13/06/2024 | 1 | Arakan or Bangladesh or Bengal or "Cox's Bazaar" or "Cox's Bazar" or Myanmar or "Rakhine State" or Rohingya | 213 |
| | | 2 | asylum or "asylum seek*" or displace* or diaspora or "Forcibly displaced Myanmar National" or enslaved or fdmn or "Forced migrat*" or ethni* or foreign* or "humanitarian crisis" or "humanitarian intervention*" or illegal or immigrant* or migra* or minorit* or "People-of-concern" or "Persons-of-concern" or refugee* or Rohingya* or separated or smuggl* or traffick* or transient* or transmigra* or undocumented | |
| | | 3 | Vaccin* or Immun* or "Immuni?ation schedule" or "vaccin* schedule" or "Immuni?ation program*" or "vaccin* program*" or "Mass vaccination" or "mass immuni?ation" or "vaccin* campaign*" or "immuni?ation campaign*" or "vaccin* refusal" or "vaccin* hesitan*" or "immuni?ation refusal" or "immuni?ation hesitancy" or "vaccin* cover*" or "immuni?ation cover*" or "vaccine-preventable-disease*" | |
| | | 4 | 1 and 2 and 3 | |

## Supplement 4: Information sources and search strategy of grey literature search

*Platforms:*

| | |
|---|---|
| Bangladesh Journals Online https://www.banglajol.info/ | Broad search terms such as “Rohingya Refugees” or “Forcibly Displaced Myanmar Nationals” |
| https://www.humanitarianresponse.info | ‘Bangladesh: Rohingya Refugee Crisis 2017-2022’ and ‘Assessment reports’ |
| https://reliefweb.int | ‘Bangladesh’ and ‘Health’ |

*Organizations and other:*

WHO
UNFPA
UNICEF
UNHCR
GIZ
USAID
Save the Children
UnOCHA
Red Cross
DFID
World Bank
CIDA
JICA
KFW
IOM
MSF

Websites of relevant organisations were searched with broad search terms such as ‘Rohingya Refugees’ or ‘Forcibly Displaced Myanmar Nationals’ and titles screened for grey literature.

*Google search:*

The Google search was undertaken after clearing previous history and cookies. The Google Advanced Search Function was used with the key concepts (Rohingya Refugees’ or ‘Forcibly Displaced Myanmar Nationals") and (immunisation or vaccination) and (Cox's Bazaar or Cox's Bazar) and titles of the first 10 pages screen

# Supplement 5: Overview of included literature

## Supplement 5.1 Overview of peer-reviewed literature

| **Author, Year** | **Evidence Source** | **Summary** | **Context** | **Population** | **Target Behaviour** | **COM-Factor Barrier/ Driver** | **Intervention Type** | **Critical Appraisal** (Quality criteria met/ Total) |
|---|---|---|---|---|---|---|---|---|
| **Ahmed 2023 (3)** | Primary research | Cross sectional study of 224 FDMN/ RR caregivers using convenience sampling and interviewer-administered semi-structured questionnaires to explore factors behind low vaccination uptake. | Registered camps: Kutapulong and Nayapara Unregistered / Makeshift camps: 7, 26 | FDMN/ RR | Receiving | • Capability<br>• Motivation<br>• Social Opportunity<br>• Physical<br>• Opportunity | • Environmental restructuring | 7/7 |
| **Chan, 2018 (4)** | Opinion piece | Article reviewing evidence in published literature on main health risks associated with communicable diseases for RR/FDMN and vaccination campaigns from 2017 | Ukhiya, Teknaf subdistricts | HSP | Facilitating | • Physical Opportunity | • Environmental restructuring | 6/6 |
| **Feldstein, 2020 (5)** | Primary research | Cross-sectional serologic and self-reported vaccination coverage survey from April-May 2018 among 930 children via caregiver interview. Simple random sampling of household lists for Nayapara and cluster sampling for Makeshift camp | Nayapara, Makeshift camps | FDMN/RR | Receiving Facilitating | • Social Opportunity<br>• Physical Opportunity<br>• Motivation | • Environmental restructuring | 6/7 |
| **Hsan, 2019 (6)** | Opinion piece | Letter to editor about varicella outbreak from December 2018 - January 2019 in Cox’s Bazar and prevention strategies | Cox's Bazar | FDMN/RR | Receiving | • Motivation | • Environmental restructuring | 5/6 |

| | | | | | | | | |
|---|---|---|---|---|---|---|---|---|
| **Hsan, 2020 (7)** | Opinion piece | Letter to editor about diphtheria outbreak and vaccination campaigns from December 2017-March 2018 | Cox's Bazar | FDMN/RR | Receiving Facilitating | • Physical Opportunity<br>• Motivation | • Environmental restructuring | 5/6 |
| **Jalloh, 2019 (8)** | Primary research | Qualitative research from January 2018 on vaccination barriers and opportunities for diphtheria and other vaccinations following vaccination campaign in December 2017. Purposeful and door-to-door sampling. Included nine focus group discussions (FGD) and 15 interviews with community and religious leaders, and caregivers/parents of children <5 years. | Balukhali, Hakimpara, Kutupalong camps | FDMN/RR | Receiving Facilitating | • Capability<br>• Social Opportunity<br>• Physical Opportunity<br>• Motivation | • Information/ Education<br>• Environmental restructuring<br>• Modelling | 7/7 |
| **Jalloh, 2020 (9)** | Opinion piece | Review article on three examples of social mobilisation in low-income settings including Cox's Bazar from 2018, based on previous research article by Jalloh et al. | Cox's Bazar | FDMN/RR | Receiving | • Social Opportunity<br>• Physical Opportunity | • Information/ Education<br>• Persuasion<br>• Training<br>• Environmental restructuring<br>• Modelling | 5/6 |
| **Khan 2019 (10)** | Primary research | Cross-sectional self-report survey and observer recorded data to evaluate oral cholera, polio, intramuscular measles, and rubella vaccination campaign undertaken Sep-Oct 2017. 92 cluster randomly surveyed of population (N= 39,483 from target population N=40,779) | Balukhali camps, 4 major settlements | FDMN/RR | Receiving Facilitating | • Capability<br>• Social Opportunity<br>• Physical Opportunity<br>• Motivation | • Environmental restructuring<br>• Training<br>• | 6/7 |
| **Khan 2023 (11)** | Review | Review of seven rounds OCV campaigns between October 2017-December 2021. Describes vaccination campaigns high level of vaccine coverage between 87-108% in FDMN and host communities | Ukhiya and Teknaf subdistricts | FDMN | Receiving | / | • Information/ Education<br>• Environmental restructuring | 5/6 |
| **Qadri, 2018 (12)** | Opinion piece | Report on oral cholera vaccination campaign in 2017 using single dose regime for adults and children over 5 years | 155 vaccination sites in 4 major settlements- (not further detailed) | Both | Facilitating | • Physical Opportunity | • Environmental restructuring<br>• Modelling<br>• Persuasion | 5/6 |

| | | | | | | | | |
|---|---|---|---|---|---|---|---|---|
| **Qayum, 2023 (13)** | Primary research | Vaccine coverage evaluation survey following oral cholera vaccination (OCV) campaign 6-13th May 2018. Involved cross-sectional survey of N=4240 using multistage cluster sampling in host community, registered and makeshift camps via interviews and vaccination card review. Overall OCV coverage 85%, 68% host community, 98% makeshift camps | Ukhyia subdistrict: Kutapulong registered camp, makeshift camps. Teknaf subdistrict: host community | FDMN/ RR | Receiving | • Capability<br>• Motivation<br>• Physical Opportunity | • Environmental restructuring | 5/6 |
| **Rahman, 2019 (14)** | Opinion piece | Report on three diphtheria vaccination campaigns in Jan-Feb 2018. | Cox's Bazar | FDMN/RR | Receiving | • Motivation | • Environmental restructuring | 5/6 |
| **Summers, 2018 (15)** | Primary research | Three cross-sectional self-report surveys following OCV campaigns in Oct-Nov 2017. Households selected using simple random sampling in Kutapalong (N=309) and Nayapara (N=408) and via multistage cluster sampling in makeshift camps (N= 1110). Also reported on pentavalent vaccine campaign in December targeting children 6 months -6 years, though no further details given | Kutapalong, Nayapara, Makeshift camp | FDMN/RR | Receiving<br>Facilitating | • Social Opportunity<br>• Physical Opportunity | • Environmental restructuring | 6/7 |

## Supplement 5.2 Overview of grey literature

| Author, Year | Evidence Source | Summary | Context | Population | Target Behaviour | COM-Factor | Intervention Type | Critical Appraisal (Quality Criteria Met/ Total) |
|---|---|---|---|---|---|---|---|---|
| **Bangladesh Health Watch 2019 (16)** | NGO report | Literature review on eight key health issues and description of cholera and diphtheria vaccination campaigns in 2018 | Cox's Bazar | Both | Receiving | • Motivation | • Environmental restructuring<br>• Persuasion<br>• Modelling | 22/29 |
| **BBC Media Action, Translators without Borders, 2019 (17)** | NGO primary research | Focus group discussion in September 2019 with male and female participants (aged 18-25 and over 26 years). | Camp 2 | FDMN/RR | Receiving | • Capability<br>• Social Opportunity<br>• Motivation | | 16/24 |
| **BRAC, 2019 (18)** | NGO primary research | Mixed method study with random selection of 36 households close to BRAC health facilities from 2018-April 2019<br>Quantitative: cross-sectional household self-report survey<br>Qualitative: 12 interviews, 2 FGD in high- and low-density camp, Delphi study with 22 service providers | Quantitative: Camps 1E,7,9 8E, 11, 13, 14, 15, 16, 22; Qualitative: camps 1E and 8E | Both | Receiving | • Capability<br>• Social Opportunity<br>• Physical Opportunity | • Environmental restructuring | 28/30 |
| **Red R India, 2018 (19)** | NGO response evaluation report | Mixed method NGO response evaluation from 2018.<br>Quantitative: secondary data review including observer recorded data.<br>Qualitative: interviews with service providers, government officials, FGD with FDMN/RR, case study and household self-report survey. Purposeful sampling of participants | Qualitative method: Camp 12, 13, 15, 18 and 19 | Both | Receiving | • Capability | • Environmental restructuring | 22/30 |
| **UNHCR, 2018 (20)** | UN agency response evaluation report | Mixed methods evaluation of UN agency response between August 2017-September 2018. Qualitative: 120 staff interviews; 26 FDMN/RR interviews; 30 FGD with FMDN/RR, community leaders, UN/NGO officials; secondary data review including observer recorded data. Quantitative: secondary data analysis; fresh analysis of 10 existing cross-sectional self-report and geographical survey data sets | Camp 1, 2, 4 (Extension), 11, 18, 20 (Extension), Kutupalong Registered, Nayapara Registered, Leda, 24, Shalbon Jadimura, 27 | Both | Receiving<br>Facilitating | • Physical Opportunity | • Environmental restructuring | 28/30 |

| | | | | | | | | |
|---|---|---|---|---|---|---|---|---|
| **UNICEF, 2018 (21)** | UN agency response evaluation report | Evaluation study using retrospective and formative evaluations from March-October 2018<br>Quantitative: documentary review including observer recorded data.<br>Qualitative: documentary review; interviews; 13 FGD; two online self-report surveys | Cox's Bazar | Both | Receiving Facilitating | • Social Opportunity<br>• Physical Opportunity | • Information/ Education<br>• Environmental restructuring | 28/30 |
| **UNICEF, 2020 (22)** | UN agency situation report | End of Year report from 2020 including situation overview, humanitarian needs and summary analysis of programme response | Cox's Bazar | Both | Receiving Facilitating | • Capability<br>• Physical Opportunity | • Environmental restructuring | 15/23 |
| **SEARO WHO, 2019 (23)** | UN agency report | Literature review summarising Rohingya crisis and health efforts, reporting on vaccination campaigns from October 2017 till initiation of routine immunization in February 2018 | Cox's Bazar | HSP | Receiving Facilitating | • Social Opportunity<br>• Physical Opportunity<br>• Motivation | • Information/ Education<br>• Environmental restructuring<br>• Training | 22/29 |

## Supplement 6: Critical appraisal of included articles

Legend

Yes Unclear No N/A

## Critical Appraisal of Studies using MMAT

| Author, Year | S1. Are there clear research questions? | S2. Do the collected data allow to address the research questions? | 1.1. Is the qualitative approach appropriate to answer the research question? | 1.2. Are the qualitative data collection methods adequate to address the research question? | 1.3. Are the findings adequately derived from the data? | 1.4. Is the interpretation of results sufficiently substantiated by | 1.5. Is there coherence between qualitative data sources, collection, analysis? and interpretation? | 2.1. Is randomization appropriately performed? | 2.2. Are the groups comparable at baseline? | 2.3. Are there complete outcome data? | 2.4. Are outcome assessors blinded to the intervention provided? | 2.5 Did the participants adhere to the assigned intervention? | 3.1. Are the participants representative of the target population? | 3.2. Are measurements appropriate regarding both the outcome and intervention (or exposure)? | 3.3. Are there complete outcome data? | 3.4. Are the confounders accounted for in the design and analysis? | 3.5. During the study period, is the intervention administered (or exposure occurred) as intended? | 4.1. Is the sampling strategy relevant to address the research | 4.2. Is the sample representative of the target population? | 4.3. Are the measurements appropriate? | 4.4. Is the risk of nonresponse bias low? | 4.5. Is the statistical analysis appropriate to answer the research | 5.1. Is there an adequate rationale for using a mixed methods design to address the research question? | 5.2. Are the different components of the study effectively integrated to answer the research question? | 5.3. Are the outputs of the integration of qualitative and quantitative components adequately interpreted? | 5.4. Are divergences and inconsistencies between quantitative and qualitative results adequately addressed? | 5.5. Do the different components of the study adhere to the quality criteria of each tradition of the methods involved? | **Rating** | **Percentage** |
|---|---|---|---|---|---|---|---|---|---|---|---|---|---|---|---|---|---|---|---|---|---|---|---|---|---|---|---|---|---|
| Ahmed 2023 | 🟢 | 🟢 | 🟢 | 🟢 | 🟢 | 🟢 | 🟢 | | | | | | | | | | | | | | | | | | | | | **7/7** | **100%** |
| Feldstein, 2020 | 🟢 | 🟢 | | | | | | | | | | | | | | | | 🟢 | 🟢 | 🟢 | 🔴 | 🟢 | | | | | | **6/7** | **86%** |
| Summers, 2018 | 🟢 | 🟢 | | | | | | | | | | | | | | | | 🟢 | 🟢 | 🟢 | 🟣 | 🟢 | | | | | | **6/7** | **86%** |
| Khan, 2019 | 🟢 | 🟢 | | | | | | | | | | | | | | | | 🟢 | 🟢 | 🟢 | 🟣 | 🟢 | | | | | | **6/7** | **86%** |
| Jalloh, 2019 | 🟢 | 🟢 | 🟢 | 🟢 | 🟢 | 🟢 | 🟢 | | | | | | | | | | | | | | | | | | | | | **7/7** | **100%** |
| Qayum, 2023 | 🟢 | 🟢 | | | | | | | | | | | | | | | | 🟢 | 🟢 | 🟢 | 🟣 | 🟢 | | | | | | **6/7** | **86%** |

## Critical Appraisal of Grey Literature using AACODS

| Author, Year | Individual: Associated with a reputable organisation? | Individual: Professional qualifications or considerable experience? | Individual: Produced/published other work (grey/black) in the | Individual: Recognised expert, identified in other sources? | Individual: Cited by others? (use Google Scholar as a quick check) | Individual: Higher degree student under " expert" supervision? | Organisation: Is the organisation reputable? (e.g. W.H.O) | Organisation: Is the organisation an authority in the field? | Does the item have a detailed reference list or bibliography? | Does the item have a clearly stated aim or brief? | If so, is this met? | Does it have a stated methodology? | If so, is it adhered to? | Has it been peer-reviewed? | Has it been edited by a reputable authority? | Supported by authoritative, documented references or credible | Is it representative of work in the field? | If No, is it a valid counterbalance? | Is any data collection explicit and appropriate for the research? | If item is secondary material (e.g. a policy brief of a technical report) refer to the original. Is it an accurate, unbiased | Are any limits clearly stated? | Opinion, expert or otherwise, is still opinion: is the author' s standpoint clear? | Does the work seem to be balanced in presentation? | Does the item have a clearly stated date related to content? No easily discernible date is a strong concern. | If no date is given, but can be closely ascertained, is there a valid reason for its absence? | Check the bibliography: have key contemporary material been | Is the item meaningful? (this incorporates feasibility, utility and | Does it add context? | Does it enrich or add something unique to the research? | Does it strengthen or refute a current position? | Would the research area be lesser without it? | Is it integral, representative, typical? | Does it have impact? (in the sense of influencing the work or behaviour of others) | **Quality Criteria Met** | **Percentage** |
|---|---|---|---|---|---|---|---|---|---|---|---|---|---|---|---|---|---|---|---|---|---|---|---|---|---|---|---|---|---|---|---|---|---|---|---|
| UNHCR, 2017 | Green | Green | Green | Green | Green | | Green | Green | Green | Green | Green | Green | Green | Red | Purple | Green | Green | | Green | Green | Green | Green | Green | Green | | Green | Green | Green | Green | Green | Green | Green | Green | **28/30** | **93%** |
| UNICEF, 2020 | | | | | | | Green | Green | Red | Red | | Red | | Purple | Purple | Green | Green | | Purple | Purple | Green | Green | Red | Green | | Green | Green | Green | Green | Green | Green | Green | Green | **15/23** | **65%** |
| BBC MEDIA ACTION , 2019 | | | | | | | Green | Green | Red | Green | Green | Green | Green | Red | Purple | Red | Green | | Green | | Green | Purple | Purple | Green | | Red | Green | Green | Green | Green | Green | Green | Purple | **16/24** | **67%** |
| BRAC, 2019 | Green | Green | Green | Green | Green | | Green | Green | Green | Green | Green | Green | Green | Red | Green | Green | Green | | Green | Green | Green | Purple | Green | Green | | Green | Green | Green | Green | Green | Green | Green | Green | **28/30** | **93%** |
| BHW, 2019 | Green | Green | Green | Green | Green | | Green | Green | Green | Purple | Purple | Red | | Red | Green | Green | Green | | Purple | Purple | Green | Green | Purple | Green | | Green | Green | Green | Green | Green | Green | Green | Green | **22/29** | **76%** |

| | | | | | | | | | | | | | | | | | | | | | | | | | | | | | | | | | | | |
|---|---|---|---|---|---|---|---|---|---|---|---|---|---|---|---|---|---|---|---|---|---|---|---|---|---|---|---|---|---|---|---|---|---|---|---|
| WHO SEARO, 2019 | 🟢 | 🟢 | 🟢 | 🟢 | 🟢 | | 🟢 | 🟢 | 🟢 | 🟢 | 🟢 | 🔴 | | 🔴 | 🟣 | 🟢 | 🟢 | | 🟣 | 🟣 | 🟢 | 🟢 | 🟣 | 🟢 | | 🟢 | 🟢 | 🟢 | 🟢 | 🟢 | 🟣 | 🟢 | 🟢 | **22/29** | **76%** |
| Red R India, 2019 | 🟢 | 🟢 | 🟣 | 🟣 | 🟣 | | 🟢 | 🟢 | 🔴 | 🟢 | 🟢 | 🟢 | 🟢 | 🟣 | 🟣 | 🔴 | 🟢 | | 🟢 | 🟢 | 🟢 | 🟢 | 🟢 | 🟢 | | | 🟢 | 🟢 | 🟢 | 🟢 | 🟢 | 🟢 | 🟢 | **22/29** | **76%** |
| UNICEF, 2020 | 🟢 | 🟢 | 🟢 | 🟢 | 🟢 | | 🟢 | 🟢 | 🟢 | 🟢 | 🟢 | 🟢 | 🟢 | 🟣 | 🔴 | 🟢 | 🟢 | | 🟢 | 🟢 | 🟢 | 🟢 | 🟢 | 🟢 | | 🟢 | 🟢 | 🟢 | 🟢 | 🟢 | 🟢 | 🟢 | 🟢 | **28/30** | **93%** |

***Critical Appraisal of Text and Opinion Pieces using JBI Checklist***

| Author, Year | 1. Is the source of the opinion clearly identified? | 2. Does the source of opinion have standing in the field of expertise? | 3. Are the interests of the relevant population the central focus of the opinion? | 4. Is the stated position the result of an analytical process, and is there logic in the opinion expressed? | 5. Is there reference to the extant literature? | 6. Is any incongruence with the literature/sources logically defended? | **Quality criteria met** | **Percentage of quality criteria met** |
|---|---|---|---|---|---|---|---|---|
| Chan, 2018 | 🟢 | 🟢 | 🟢 | 🟢 | 🟢 | 🟢 | **6/6** | **100%** |
| Hsan, 2020 | 🟢 | 🟢 | 🟢 | 🟢 | 🟢 | 🟣 | **5/6** | **83%** |
| Hsan, 2019 | 🟢 | 🟢 | 🟢 | 🟢 | 🟢 | 🟣 | **5/6** | **83%** |
| Jalloh, 2020 | 🟢 | 🟢 | 🟣 | 🟢 | 🟢 | 🟢 | **5/6** | **83%** |
| Khan 2023 | 🟢 | 🟢 | 🟢 | 🟢 | 🟢 | 🟣 | **5/6** | **83%** |
| Qadri, 2019 | 🟢 | 🟢 | 🟢 | 🟢 | 🟢 | 🟣 | **5/6** | **83%** |
| Rahman, 2019 | 🟢 | 🟢 | 🟢 | 🟢 | 🟢 | 🟣 | **5/6** | **83%** |

**Supplements reference list**


1. Bhatia A, Mahmud A, Fuller A, Shin R, Rahman A, Shatil T, et al. The Rohingya in Cox's Bazar: When the Stateless Seek Refuge. Health and human rights. 2018;20(2):105–22.
2. Jordan Z, Konno R, Mu PF. Synthesizing Evidence from Narrative, Text and Opinion. In: Professor Alan Pearson A, Professor of Evidence Based Healthcare and Executive Director of the, Joanna Briggs Institute; Faculty of Health Sciences at the University of Adelaide SA, editors. Synthesis Science in Healthcare Series: Book 32011.
3. Ahmed N, Ishtiak ASM, Rozars MFK, Bonna AS, Alam KMP, Hossan ME, et al. Factors associated with low childhood immunization coverage among Rohingya refugee parents in Cox's Bazar, Bangladesh. PLoS One. 2023;18(4):e0283881.
4. Chan EYY, Chiu CP, Chan GKW. Medical and health risks associated with communicable diseases of Rohingya refugees in Bangladesh 2017. Int J Infect Dis. 2018;68:39–43.
5. Feldstein LR, Bennett SD, Estivariz CF, Cooley GM, Weil L, Billah MM, et al. Vaccination coverage survey and seroprevalence among forcibly displaced Rohingya children, Cox's Bazar, Bangladesh, 2018: A cross-sectional study. PLoS medicine. 2020;17(3):e1003071.
6. Hsan K, Naher S, Gozal D, Griffiths MD, Furkan Siddique MR. Varicella outbreak among the Rohingya refugees in Bangladesh: Lessons learned and potential prevention strategies. Travel Medicine and Infectious Disease. 2019;31:101465.
7. Hsan K, Mamun MA, Misti JM, Gozal D, Griffiths MD. Diphtheria outbreak among the Rohingya refugees in Bangladesh: What strategies should be utilized for prevention and control? Travel Medicine and Infectious Disease. 2020;34:101591.
8. Jalloh MF, Bennett SD, Alam D, Kouta P, Lourenco D, Alamgir M, et al. Rapid behavioral assessment of barriers and opportunities to improve vaccination coverage among displaced Rohingyas in Bangladesh, January 2018. Vaccine. 2019;37(6):833–8.
9. Jalloh MF, Wilhelm E, Abad N, Prybylski D. Mobilize to vaccinate: lessons learned from social mobilization for immunization in low and middle-income countries. Human vaccines & immunotherapeutics. 2020;16(5):1208–14.
10. Khan AI, Islam MT, Siddique SA, Ahmed S, Sheikh N, Siddik AU, et al. Post-vaccination campaign coverage evaluation of oral cholera vaccine, oral polio vaccine and measles-rubella vaccine among Forcibly Displaced Myanmar Nationals in Bangladesh. Human vaccines & immunotherapeutics. 2019;15(12):2882–6.
11. Khan AI, Islam MT, Khan ZH, Tanvir NA, Amin MA, Khan, II, et al. Implementation and Delivery of Oral Cholera Vaccination Campaigns in Humanitarian Crisis Settings among Rohingya Myanmar nationals in Cox's Bazar, Bangladesh. Vaccines (Basel). 2023;11(4).
12. Qadri F, Azad AK, Flora MS, Khan AI, Islam MT, Nair GB, et al. Emergency deployment of oral cholera vaccine for the Rohingya in Bangladesh. Lancet (London, England). 2018;391(10133):1877–9.
13. Qayum MO, Billah MM, Sarker MFR, Alamgir ASM, Nurunnahar M, Khan MH, et al. Oral cholera vaccine coverage evaluation survey: Forcibly Displaced Myanmar Nationals and host community in Cox's Bazar, Bangladesh. Front Public Health. 2023;11:1147563.
14. Rahman MR, Islam K. Massive diphtheria outbreak among Rohingya refugees: lessons learnt. Journal of Travel Medicine. 2019;26(1).
15. Summers A, Humphreys A, Leidman E, Mil LTv, Wilkinson C, Narayan A, et al. Diarrhea and acute respiratory infection, oral cholera vaccination coverage, and care-seeking behaviors of Rohingya refugees - Cox's Bazar, Bangladesh, October-November 2017. Morbidity and Mortality Weekly Report. 2018;67(18):533–5.
16. Bangladesh Health Watch. Humanitarian Crisis in Rohingya Camps A Health Perspective. 2018–2019 (2019). https://app.bangladeshhealthwatch.org/docs/reports_pdf/bhw-reports/bangladesh-health-watch-report-2018-2019-1643129470.pdf [Accessed August 30, 2021]
17. BBC Media Action, Translators without Borders. What-Matters-Humanitarian Feedback-Bulletin Spread of misinformation in the Rohingya camps (2019).

https://translatorswithoutborders.org/wp-content/uploads/2021/11/What-Matters-Humanitarian-Feedback-Bulletin_Issue_30_English.pdf [Accessed August 30, 2021]
18. Nasar KS, Akhter I, Khaled N, Hossain MR, Majid T, Tamanna S, et al. Health Care Needs and Health Seeking Behaviour among Rohingya Refugees in Cox's Bazar: Study in selected camps served by BRAC's health care facilities (2019). https://bracjpgsph.org/assets/pdf/research/research-reports/Health-Care-Needs-and-Health_October-2019.pdf [Accessed August 30, 2021]
19. Red R India. Integrated Emergency Humanitarian Response to the Rohingya Population in Cox's Bazar- Endline Evaluation Report (2018). https://admin.concern.net/sites/default/files/documents/2020-09/Bangladesh%20ENDLINE%20EVALUATION%20REPORT%20.pdf [Accessed August 30, 2021]
20. United Nations High Commissioner for Refugees. Independent evaluation of UNHCR's emergency response to the Rohingya refugees influx in Bangladesh August 2017-2018. (2018). https://www.unhcr.org/sites/default/files/legacy-pdf/5c811b464.pdf [Accessed June 15, 2021]
21. United Nations Children's Fund. Evaluation of UNICEF's Response to the Rohingya Refugee Crisis in Bangladesh (2018). https://www.unevaluation.org/member_publications/evaluation-unicefs-response-rohingya-refugee-crisis-bangladesh [Accessed August 30, 2021]
22. United Nations Children's Fund. Bangladesh Humanitarian Situation Report No. 55 End of the Year 2020 (2020). https://www.unicef.org/documents/bangladesh-humanitarian-situation-report-end-year-2020 [Accessed August 30, 2021]
23. World Health Organzation South East Asia Regional Office. Invisible- The Rohingyas, the crisis, the people and their health (2019). https://www.who.int/publications/i/item/9789290227243 [Accessed August 30, 2021].
